# 2A self-cleaving peptide-based multi-gene expression system in the silkworm *Bombyx mori*

**DOI:** 10.1038/srep16273

**Published:** 2015-11-05

**Authors:** Yuancheng Wang, Feng Wang, Riyuan Wang, Ping Zhao, Qingyou Xia

**Affiliations:** 1 State key laboratory of silkworm genome biology, Southwest University, Chongqing, China; 2 College of biology and technology, Southwest University, Chongqing, China

## Abstract

Fundamental and applied studies of silkworms have entered the functional genomics era. Here, we report a multi-gene expression system (MGES) based on 2A self-cleaving peptide (2A), which regulates the simultaneous expression and cleavage of multiple gene targets in the silk gland of transgenic silkworms. First, a glycine-serine-glycine spacer (GSG) was found to significantly improve the cleavage efficiency of 2A. Then, the cleavage efficiency of six types of 2As with GSG was analyzed. The shortest porcine teschovirus-1 2A (P2A-GSG) exhibited the highest cleavage efficiency in all insect cell lines that we tested. Next, P2A-GSG successfully cleaved the artificial human serum albumin (66 kDa) linked with human acidic fibroblast growth factor (20.2 kDa) fusion genes and vitellogenin receptor fragment (196 kD) of silkworm linked with EGFP fusion genes, importantly, vitellogenin receptor protein was secreted to the outside of cells. Furthermore, P2A-GSG successfully mediated the simultaneous expression and cleavage of a DsRed and EGFP fusion gene in silk glands and caused secretion into the cocoon of transgenic silkworms using our sericin1 expression system. We predicted that the MGES would be an efficient tool for gene function research and innovative research on various functional silk materials in medicine, cosmetics, and other biomedical areas.

The development of new technology and research methods has brought fundamental and applied studies of the silkworm, *Bombyx mori*, into the functional genomics era^1^,^2^. Establishment of a multi-genes expression system (MGES) in silkworms allows the following potential advances: (i) visually studying the function of targeted genes, especially relationships within multiple genes (e.g. transcription factors or enzymes), (ii) simplifying the co-expression of multiple genes or multi-subunit protein complexes in silk gland bioreactor research (e.g. genes with multiple functions or antibody), (iii) creating a new multi-resistant transgenic silkworm strain (e.g. *B. mori* nuclear polyhedrosis virus (BmNPV) and cytoplasmic polyhedrosis virus (BmCPV) multi-resistant silkworm), and (iv) genetically modifying the middle and posterior silk gland of silkworms to produce multi-functional silk materials.

The 2A self-cleaving peptide (2A), which was discovered in the foot-and-mouth-disease virus (FMDV) in 1991, is an oligopeptide (usually 19–22 amino acids) located between two proteins in some members of the picornavirus family^3^. The 2A self-cleaving peptide of FMDV might undergo self-cleavage to generate mature viral proteins by a translational effect that is known as “stop-go” or “stop-carry”. The cleavage site is located between the last glycine of its C-terminal and the first praline of the 2B downstream protein (−LLNFDLLKLAGDVESNPG↓P-)^4^,^5^ (Fig. 1A). To date, 2A-like sequences in other viral mRNA molecules have been successfully identified, including the porcine teschovirus-1 2A (P2A), thosea asigna virus 2A (T2A), equine rhinitis A virus 2A (E2A), cytoplasmic polyhedrosis virus (BmCPV 2A), and flacherie virus (BmIFV 2A) of *B. mori*^6^,^7^. The MGES based on 2As has the following advantages: (i) multiple proteins known to be expressed in equivocal amounts in the same cells and tissues because they were controlled by only one promoter, (ii) 2A is small, which can readily cleave multiple proteins while minimizing the possibility of their loss of function, and (iii) proteins linked by 2A could be co-expressed in all cell types because cleavage activity was only dependent on the ribosome, which is highly structurally conserved in eukaryotes^8^. Thus, MGES based on 2A sequences have been widely applied in gene therapy to treat cancer and other diseases^6^,^9^,^10^, and to genetically modify organisms to create new functional or resistant plants and animals^11^,^12^. Furthermore, many researchers have used 2A sequences to study the function of genes in mice^13^, zebrafish^14^, swine^15^, fruit fly^16^, and sheep species^17^.

In this present study, we attempted to construct an MGES based on 2A self-cleaving peptide for functional and applied studies of the silkworm, *B. mori*. First, we confirmed that a GSG spacer could improve the cleavage efficiency of native 2A. Then, P2A with an extra GSG spacer at the N-terminus (P2A-GSG) was found to have the highest cleavage efficiency among the six types of 2As and the practicality and universality of P2A-GSG cleavage was proved through cleaving the other two artificial fusion genes linked by P2A-GSG. Furthermore, we investigated whether the selected P2A-GSG could efficiently cleave targets if linked to two fluorescent proteins in the silk gland of transgenic silkworms. The findings of this study will guide the effective design of multi-gene expression vectors and promote innovative fundamental and material studies of silkworms and *Lepidopteran* insects, as well as other species in the future.

## Results

**Figure 1:**
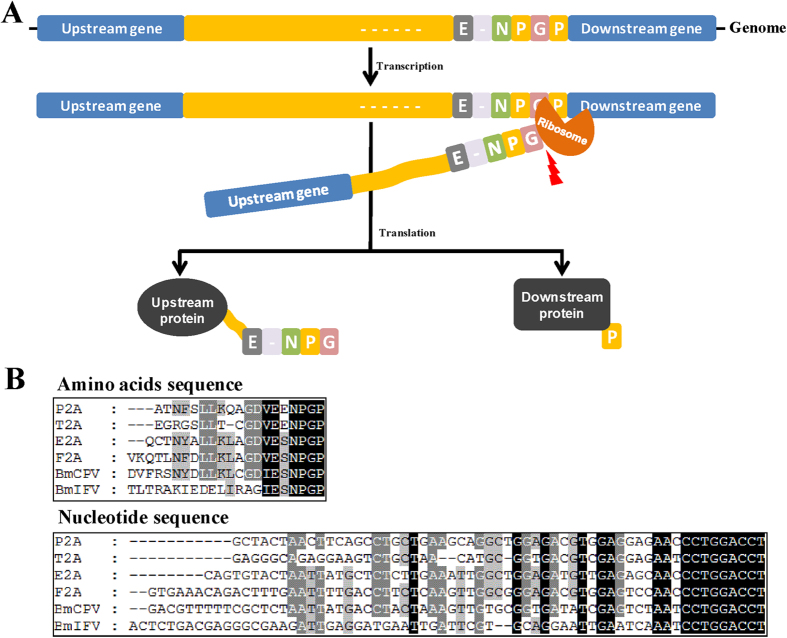
Cleavage mechanism and sequence analysis of 2A self-cleaving peptide. (**A**) Self-cleavage mechanism of 2A self-cleaving peptide. The cleavage site locates between glycine (G) and praline (P) at its C-terminus. After self-cleavage, amino acids residues at the N-terminus of 2A linked to upstream protein and its last praline residue remained to the downstream protein, but the residues of 2A could be removed through furin and signal peptide^9^. (**B**) Sequence analysis of six 2As which was respectively from porcine teschovirus-1 2A (P2A), thoseaasigna virus 2A (T2A), equine rhinitis a virus 2A (E2A), foot and mouth disease virus 2A (F2A), cytoplasmic polyhedrosis virus 2A (BmCPV2A) and flacherie Virus 2A (BmIFV2A).

**Figure 2:**
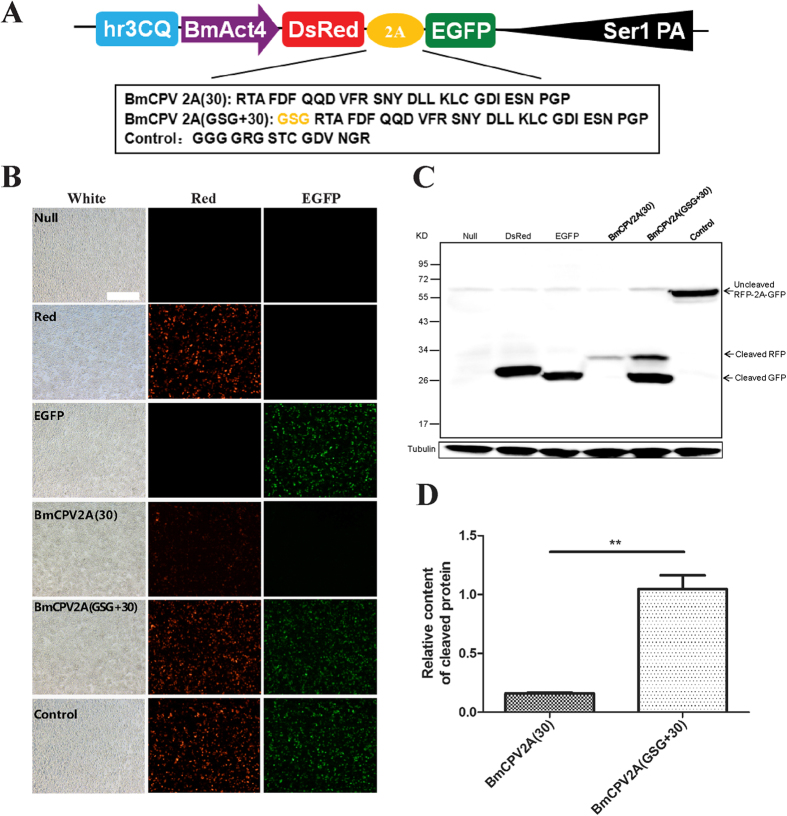
GSG spacer significantly improved the cleavage efficiency of 2A self-cleaving peptide in Sf9 cell. (**A**) Schematic diagram of DsRed-2A-EGFP fusion gene expression vectors. BmCPV2A(30)^8^, BmCPV2A(GSG+30) and Control respectively was the native BmCPV2A, GSG spacer added BmCPV2A(30) and negative control. The skeleton of expression vector contained hr3CQ enhancer, BmAct4 promoter and Ser1PA was used by Wang^22^ (the same below). (**B**) The results of fluorescent signal post-transfection in the Sf9 cell. The White, Red and EGFP respectively indicated the results in the white, red and green lights (the same below). (**C**) Protein analysis of samples from the Sf9 cells. (**D**) Cleavage efficiency analysis between BmCPV2A (30) and BmCPV2A(GSG+30) in the Sf9 cell. Scale bar, 400um.

To screen for the 2A self-cleaving peptide with the highest cleavage efficiency and shortest amino acid length, we first aligned and analyzed the nucleic acid and amino acid sequences of four commonly used 2A self-cleaving peptides: P2A, T2A, E2A, F2A, and BmCPV2A, BmIFV2A which are from viruses that can infect silkworm larvae^7^ (Fig. 1B). A highly conserved region in the C-terminus of six selected 2As was identified, which was involved in self-cleavage according to previous reports^4^,^5^. Then, we analyzed whether a glycine-serine-glycine spacer (GSG)^6^,^18^,^19^ fused at the N-terminus of 2A could improve its cleavage efficiency for BmCPV2A (30)^7^, because our previous data showed that native BmCPV2A (30) exhibited a low cleavage efficiency in Sf9 and BmE cell lines. A total of three fusion-gene expression vectors based on hr3CQ usage of enhanced BmAct4 promoter were constructed and were transiently expressed in insect-derived cell lines (Fig. 2A). Both the RFP and EGFP fluorescence signals could be detected in BmCPV2A (30), BmCPV2A (GSG+30), and negative control vector-transfected Sf9 and BmNS cells (Fig. 2B, Supplementary Figure 1A), suggesting that hr3CQ enhanced BmAct4 promoter-driven efficient expression of target genes. The cleaved immunoblot bands of RFP (~30 kDa) and GFP (~28 kDa), indicated by reactivity with specific antibodies, could be detected in BmCPV2A (30) and BmCPV2A (GSG+30) vector, but not in negative control vector-transfected cells, suggesting that BmCPV2A (30) and BmCPV2A (GSG+30) functioned to cleave and generate mature RFP and EGFP (Fig. 2C, Supplementary Figure 1B). Based on band intensity, the cleavage of RFP and GFP by BmCPV2A (GSG+30) was enhanced 6.58-fold in Sf9 cells and 1.69-fold in BmNS cell compared with that by BmCPV2A (30), besides, both RFP and EGFP fluorescence signals intensity in the BmCPV2A(GSG+30) were much stronger than that in BmCPV2A(30) (Fig. 2B, Supplementary Figure 1A), which indicated that the addition of a GSG spacer at the N-terminus significantly improved the cleavage efficiency of BmCPV2A (30) (Fig. 2D, Supplementary Figure 1C).

**Figure 3:**
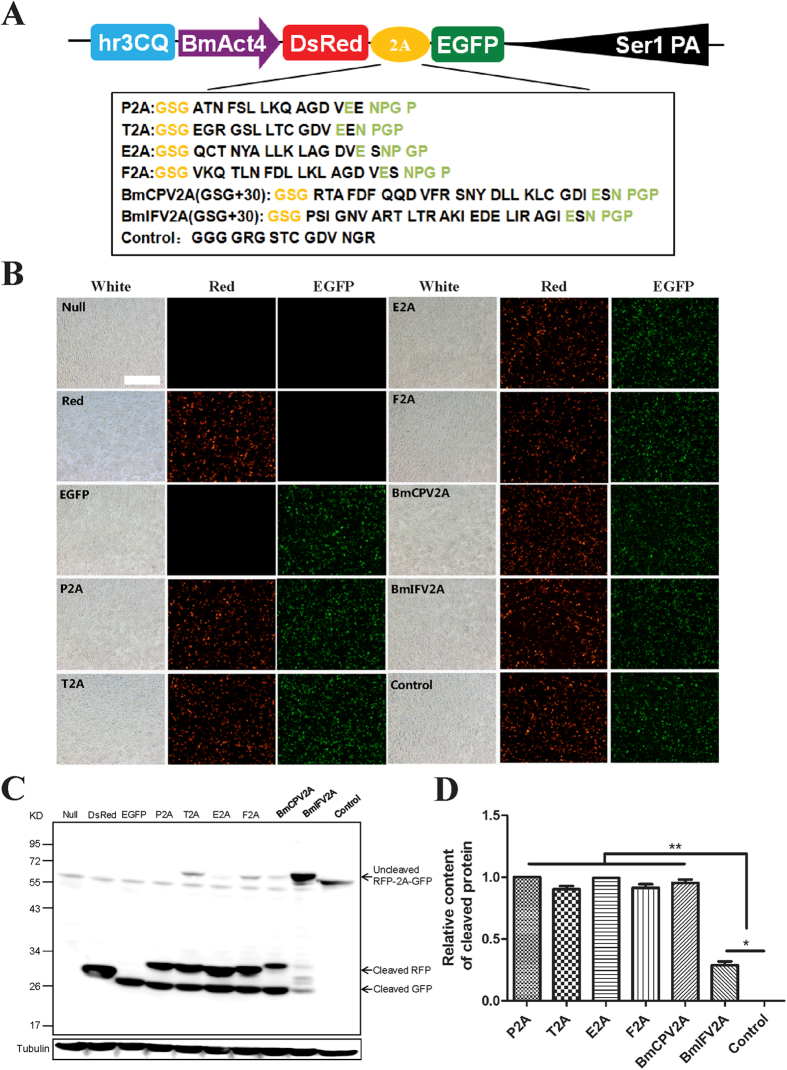
P2A-GSG possesses the highest cleavage efficiency to cleave DeRed-2A-EGFP fusion gene in the Sf9 cell. (**A**) Schematic diagram of DsRed-2A-EGFP fusion gene expression vectors. GSG spacer was added at the N-terminus of each P2A, T2A, E2A, F2A, BmCPV2A and BmIFV2A. (**B**) The results of fluorescent signal post-transfection in the Sf9 cells. (**C**) Protein analysis of samples from the Sf9 cells. (**D**) Cleavage efficiency analysis of six type 2A self-cleaving peptides in the Sf9 cells. Scale bar, 400 um.

Next, a GSG spacer was added at the N-terminus of six types of 2As: P2A, T2A, E2A, F2A, BmIFV2A, and BmCPV2A; their cleavage efficiencies were analyzed by transient expression (Fig. 3A). Both RFP and GFP fluorescent signals and cleaved RFP (~30 kDa) and GFP (~28 kDa) protein immunoblot bands could be detected in all 2A-GSGs vector-transfected cells, suggesting that all of the 2A-GSGs functioned in Sf9 cells (Fig. 3B,C). Among these 2A-GSGs, we found that P2A-GSG possessed the highest cleavage efficiency, which completely cleaved the DsRed-EGFP fusion gene product (100%). Additionally, E2A-GSG, T2A-GSG, F2A-GSG, and BmCPV2A-GSG could cleave most of the fusion genes (>90%), which was significantly higher than that of BmIFV2A-GSG (~29%) (Fig. 3D). Similar results were also obtained in transfected BmE and BmNS cells (Supplementary Figure 2). These results above suggested that the P2A-GSG with the highest cleavage efficiency and shortest length could be the best candidate for an MGES in silkworms (Table 1).

**Figure 4:**
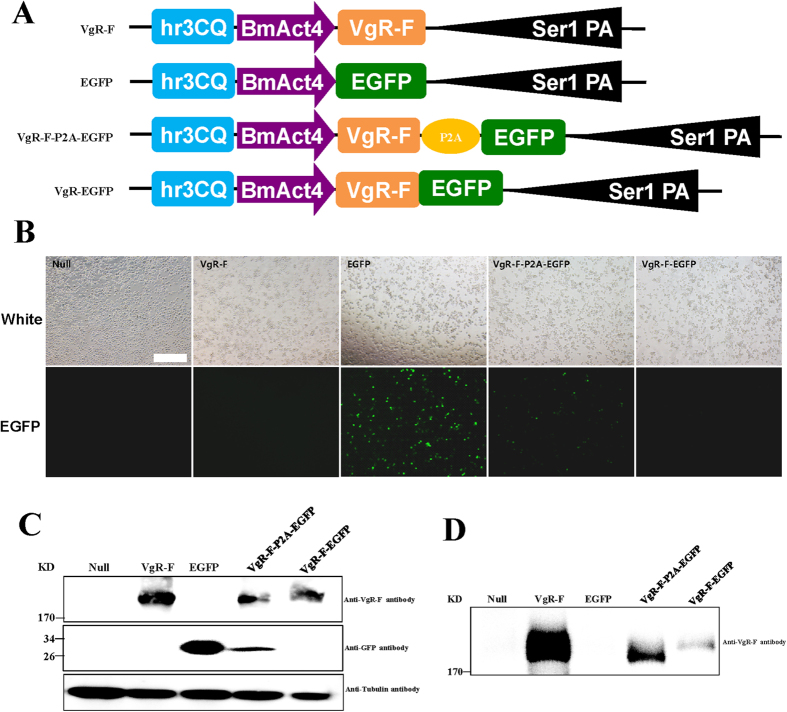
P2A-GSG cleaved the VgR-F-2A-EGFP fusion gene in the Sf9 cell. (**A**) Schematic diagram of VgR-F, EGFP, VgR-F-P2A-EGFP and VgR-F-EGFP fusion gene expression vectors. (**B**) The results of fluorescent signal post-transfection in the Sf9 cells. (**C**) Protein analysis of samples from the Sf9 cells. (**D**) Protein analysis of samples from the culture mediums of Sf9 cells. Scale bar, 400 um.

The practicality and universality of P2A-GSG to cleave other customized fusion genes with different molecular weights were further analyzed. First, human serum albumin (HSA, molecular weight 66 kDa) and human acidic fibroblast growth factor (FGF1, molecular weight 20.2 kDa) with two types of sort orders were both linked by P2A-GSG (Supplementary Figure 3A) and could be cleaved into the HSA-2A plus FGF1, FGF1-2A plus HSA protein products with corresponding molecular weights (Supplementary Figure 3B and 3C) in BmE and BmNS cells, suggesting the cleavage mediated by P2A-GSG was not affected by the gene sort orders. Then, a relative large protein vitellogenin receptor fragment (VgR-F, 196kD)^20^ of silkworm was linked to EGFP with P2A-GSG (Fig. 4A) and these expression vectors were transfected to two insect-derived Sf9 and BmE cells. Obvious EGFP fluorescence signals were observed after the EGFP and VgR-F-P2A-EGFP post-transfection, but not in the VgR-F-EGFP (Fig. 4B), showing that P2A-GSG cleaved the VgR-F and EGFP fusion gene. The cleaved products of the VgR-F, EGFP were also detected in the Sf9 and BmE cells (Fig. 4C, Supplementary Figure 4A), which indicated that the linked large protein VgR-F by P2A-GSG could be also cleaved and the practicality and universality of P2A-GSG for the co-expression of various fusion genes. Importantly, the cleaved VgR-F was detected in the culture mediums of Sf9 and BmE cells (Fig. 4D, Supplementary Figure 4B), suggesting that the cleaved VgR-F was secreted as the same with native VgR-F and cleavage of P2A-GSG could not affect the target’s function.

**Figure 5:**
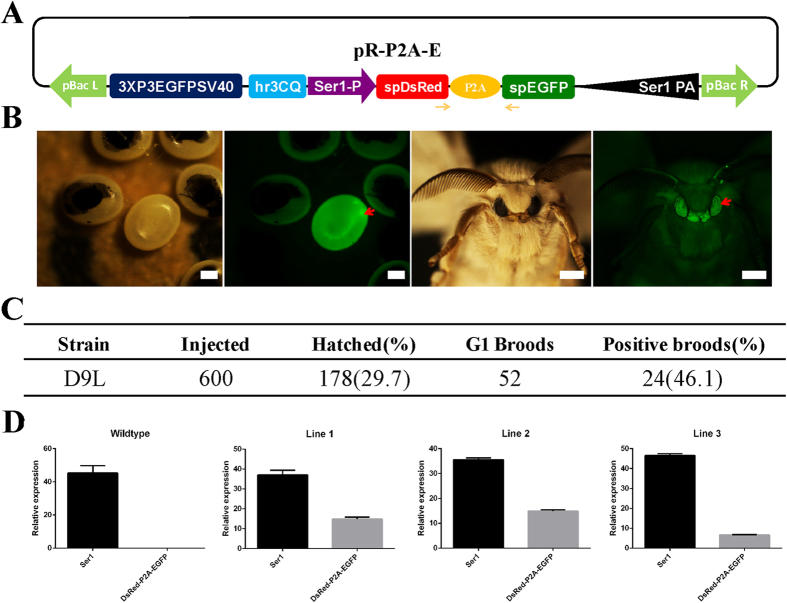
Generation of transgenic spDsRed-P2A-spEGFP silkworm. (**A**) Schematic diagram of pR-P2A-E transgenic vector. spDsRed-P2A-spEGFP was fusion gene consisted of signal peptide of Ser1 gene (sp), red fluorescent protein gene (DsRed), enhanced green fluorescent protein gene (EGFP) and P2A-GSG self-cleaving peptide. The skeleton of expression vector contained hr3CQ enhancer, Ser1-P promoter, Ser1PA, 3xp3EGFPSV40, pBacL and pBacR were used by Wang^22^. (**B**) Screen for positive transgenic silkworm. (**C**) Microinjection analysis of the transgenic spDsRed-P2A-spEGFP silkworm. (**D**) Expression of fusion gene on mRNA level by real-time PCR. Ser1 is the endogenous gene, as a control and orange arrows were primers used in (**A**). Scale bar, 2 mM.

**Figure 6:**
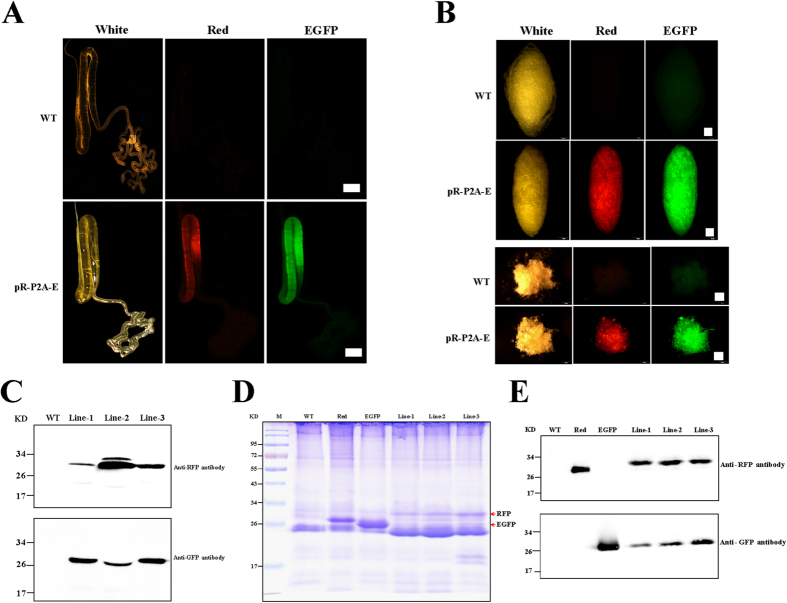
Expression of the spDsRed-P2A-spEGFP fusion gene in the transgenic silkworm. (**A**,**B**) Fluorescent images for the middle silk grand and cocoons of the transgenic spDsRed-P2A-spEGFP silkworm. WT and pR-P2A-E were the wild type and positive transgenic silkworm. (**C**) Protein analysis of samples from the middle silk grand with anti-RFP, anti-GFP antibodies. (**D**,**E**) Protein analysis for the samples from the cocoons by the coomassie brilliant blue (CCB) staining and western blotting with anti-RFP, anti-GFP antibodies. Scale bar, 2 mM.

The cleavage efficiency of P2A-GSG for the two co-expressed genes in transgenic silkworms was further analyzed using our previously constructed sericin1 expression system^21^,^22^. In this system, a fusion gene spDsRed-P2A-spEGFP, in which DsRed and EGFP each had a native ser1 signal peptide (sp) in front that was linked by P2A-GSG, was designed and inserted into a hSRSE transgenic expression vector (Fig. 5A). The purified plasmids were mixed with the hsp70-PIG helper plasmid^21^,^22^ at a 1:1 molar ratio and were microinjected into the non-diapausing eggs to generate transgenic silkworms according to a previously reported method^23^. A total of 24 broods with positive individuals were obtained by fluorescent screening of 3xp3-EGFP expression in the eyes (Fig. 5B,C), Then, the transcription of the spDsRed-P2A-spEGFP fusion gene was detected, which ranged from 14% to 40% compared to that of the endogenous ser1 gene in three randomly selected transgenic lines (Fig. 5D). To confirm whether P2A-GSG could successfully cleave spDsRed-P2A-spEGFP in transgenic silkworms, the histofluorescence property and proteins of the silk gland from spDsRed-P2A-spEGFP transgenic silkworms were analyzed. We found that the middle silk gland of spDsRed-P2A-spEGFP transgenic silkworms exhibited both RFP and GFP fluorescent signals, suggesting that the spDsRed-P2A-spEGFP fusion gene was successfully expressed by the sericin1 expression system. Furthermore, immunoblot bands of cleaved RFP (~30 kD) and GFP (~28 kD) could also be detected from proteins extracted from the silk gland of the three selected transgenic silkworm lines (Fig. 6A,C), suggesting that P2A-GSG functioned in the silkworm to cleave the spDsRed-P2A-spEGFP fusion gene. To confirm whether cleaved RFP and EGFP could be secreted into cocoons, the cocoon histofluorescence properties and transgenic expression of spDsRed-P2A-spEGFP protein in silkworms were analyzed. We found that the RFP and EGFP fluorescent signals could also be observed in cocoons (Fig. 6B) and immunoblot bands of cleaved RFP and GFP were also detected from proteins extracted from transgenic cocoons (Fig. 6D,E), suggesting that cleavage of RFP and EGFP by P2A-GSG resulted in successful secretion into the silk thread.

In summary, we successfully constructed a 2A self-cleaving peptide-based MGES for the simultaneous and efficient expression of multiple exogenous genes in the silkworm.

## Discussion

Advances in three silkworm genome sequencing projects have markedly boosted fundamental and applied studies of silkworms (*B. mori*), which also introduces higher requirements for functional genomic research technologies and platforms^1^,^2^. Because of the convenience of the simultaneous and efficient expression of multiple genes in functional research studies, the MGES based on 2A self-cleavage peptides had been widely used in various species, including cells, plants, and animals^9^,^11^,^12^,^13^,^14^,^15^,^17^, and this system will also benefit silkworm functional genomic studies.

In this present study, we attempted to construct a 2A-based MGES for silkworm functional genomic research. First, we investigated whether the selected 2A could cleave a DsRed/EGFP fusion gene, and we found that native 2A functioned inefficiently. Then, a GSG spacer was used to improve 2A cleavage by fusion at the N-terminal of 2A according to previous reports^6^,^18^,^19^. Thus, the cleavage efficiency of 2A was significantly improved. The mechanism of GSG might be that it reduces the inhibition rate of the 2A reaction by the C-terminal region of RFP (immediately upstream of 2A) when the extra GSG spacer was added^24^. Then, we compared the cleavage efficiency of six types of 2As with GSG spacers, such as P2A-GSG, E2A-GSG, BmCPV2A-GSG, T2A-GSG, F2A-GSG, and BmIFV2A-GSG, and we found that the shortest P2A-GSG possessed the highest cleavage efficiency, as it completely cleaved the DsRed/EGFP fusion gene in all insect cells tested. The cleavage efficiencies of E2A-GSG, BmCPV2A-GSG, F2A-GSG, and T2A-GSG were all over 90%, which was significantly higher than that of BmIFV2A-GSG (17%–70%). Next, we expressed the FGF1/HSA, HSA/FGF1 fusion genes and relative large protein VgR-F/EGFP fusion gene by P2A-GSG for its practicality and universality to cleave other customized fusion genes with different molecular weights. Notably, we noticed that the expression level of a downstream gene of P2A-GSG, FGF1, was clearly increased, and both FGF1 and HSA expression levels were enhanced when the HSA gene was located upstream of P2A-GSG (Supplementary Figure 3B–F). This finding was similar to that of a previous report^25^, in which mAb expression improved after transfection with an L-F2A-H expression vector compared with transfection with a H-F2A-L expression vector in CHO DG44 cells. Based on Sunghoon Hurh’s result, with increasing expression of upstream genes (e.g. hCD46, hTBM, and (HA) HO1) of 2A, corresponding downstream expression of EGFP improved^26^. We could assume that HSA should have higher expression in BmE and BmNs cells compared with FGF1, which indicated to us that gene arrangement would be an important factor in the design of an effective MGES. Moreover, the cleaved VgR-F by P2A-GSG was secreted into culture mediums as the same with native VgR-F (Fig. 4D, Supplementary Figure 4B), which showed that the cleaved VgR-F probably retained its biologic function and P2A-GSG may be an efficient tool to study gene’s function since cleavage of P2A-GSG did not affect target’s function.

Additionally, the P2A-GSG-linked spDsRed-P2A-spEGFP fusion gene, which was controlled by a previously reported sericin1 expression system, was used to generate transgenic silkworms. Both the middle silk gland and cocoon of transgenic silkworms exhibited intense DsRed and EGFP fluorescent signals, especially the immunoblot bands of cleaved fluorescent protein products that could also be detected in both the middle silk gland and cocoon of the transgenic silkworms, suggesting that P2A-GSG functioned in transgenic silkworms to cleave the linked fusion genes. These findings suggested that we successfully constructed a P2A-GSG-based MGES for simultaneous and efficient expression of multiple exogenous genes in silkworms.

The MGES will accelerate fundamental and applied silkworm research. For example, functional genes related to silkworm development and metamorphosis could be simultaneously expressed with their potential effectors or a fluorescent reporter to better understand their interactions or expression patterns. We had genetically engineered a silk with wound healing function by a transgenic silkworm^21^. Thus, silk spun by the silkworm will be simultaneously generated with two or more extra valuable functions, such as wound healing and antibacterial activity, biocompatible and tissue regenerative capacity, or high strength and toughness by MGES mediated co-expression of several functional genes, which will surely expand the applications of silk materials. Silkworms with high resistance or multiple resistances to pathogenic micro-organisms, such as BmNPV, BmCPV, and *Bombyx B. mori* densovirus (BmDPV), could be genetically modified by simultaneously expressing several antiviral genes, such as hycu-ep32 or Bmlipase-1 using the MGES^27^,^28^,^29^,^30^. Overall, our findings will establish a novel technological strategy for multi-gene expression in silkworms and will benefit functional genetic research, and could potentially allow genetic modifications of silkworms. Moreover, it will also guide the construction of MGES for other *Lepidopteran* insects and species.

## Methods

### Cell lines

The Sf9 ovary cell line from *Spodoptera frugiperda*, the BmE embryonic cell line from *B. mori*, and the BmNS ovarian cell line from *B. mori* larvae were all maintained in our laboratory. The Sf9 and BmE cell lines were maintained in Grace’s medium (Gibco), and the BmNS cell line was maintained in Tc100 medium (US Biological) supplemented with 10% fetal bovine serum (Gibco).

### Vector construction

DsRed-2A-EGFP, FGF1-P2A-HSA, and HSA-P2A-FGF1 fusion genes were amplified by overlapping PCR^9^, as described previously with slight modifications. The resulting fusion fragments were cloned into a pUC19 vector (Takara) and sequenced. Then, the correct fragments were sub-cloned into the transient expression vector pSL1180 [hr3CQ BmAct4 Luc Ser1PA]^22^,^31^, which consisted of hr3 enhancer from the nuclear polyhedrosis virus of Chongqing Bombyx mori strain (hr3CQ), the *actin 4* gene promoter from silkworm genomic DNA (BmAct4) and the 3′-UTR of the *sericin1* gene (Ser1PA); the resulting vectors were termed pSL1180 [hr3CQ BmAct4 fusion genes Ser1PA]. Then, P2A-EGFP and EGFP fragments with XhoI and NotI was amplified from pSL1180 [hr3CQBmAct4DsRed-P2A-EGFPSer1PA] and then sub-cloned into pSL1180[hr3CQBmAct4VgRSer1PA] (kindly gift from Dr. Enxiang Chen in our laboratory), thus, the resulting vectors were pSL[hr3CQBmAct4VgR-F-P2A-EGFPSer1PA] and pSL[hr3CQBmAct4VgR-F-EGFPSer1PA].

The fusion gene spDsRed-P2A-spEGFP (commercially synthesized by Genscript) encoded a P2A self-cleaving peptide, DsRed, and EGFP genes, and signal peptide from the ser1 gene (sp) was added at the N-terminus of the DsRed and EGFP genes. The spDsRed-P2A-spEGFP fragment was sub-cloned into the intermediate vector pSL1180 [hr3CQSer1DsRedSer1PA]^22^, which contained the hr3CQ enhancer, the Ser1 promoter of gene Ser1 (Ser1-P), and the Ser1PA. Then, the whole transcriptional regulation unit was sub-cloned into the pBac [3xp3EGFPaf] basic transgenic vector^23^,^32^; the resulting vector was designated pR-P2A-E. Sequences of all primers used to amplify the fusion genes are summarized in Supplementary Table 1.

### Transient expression of vectors in cells

The transfection method was based on previous reports with slight modifications^22^. Plasmids were prepared using Plasmid Mini kits (Qiagen). SF9, BmE, and BmNS cells were equivalently transfected in six-well culture plates with 1 mg of each plasmid DNA and 3 μl X-tremeGENE HP DNA Transfection Reagent (Roche) in a final volume of 500 μl antibiotic-free serum-free medium. After 5 h, medium was replaced with complete medium for 3 days.

### Generation of transgenic silkworm

The *B. mori* strain Dazao, which was reared on fresh mulberry leaves, was used in our study. Injected embryos were prepared as described in a previous protocol^33^ and the piggyBac transgenic vector pR-P2A-E mixed with a hsp70-PIG helper^34^ was microinjected into silkworm embryos 2 h after oviposition using a TransferMan NK2 micromanipulator and a Femto Jet 5247 microinjector (Eppendorf) under a SZX16 microscope (Olympus). Then, injected embryos were incubated at 25 °C and 90% relative humidity. The hatched larvae were collected and reared on fresh mulberry leaves and G0 moths were sibling crossed to screen the positive broods in G1 embryos using an Olympus SZX12 fluorescent stereomicroscope (Olympus).

### Real-time PCR

All samples were extracted from the middle silk gland of three randomly selected silkworms on the seventh day of the fifth instar. The relative expression levels of the fusion gene mRNA were quantified on an ABI Prism 7000 sequence detection system (Applied Biosystems) with a SYBR Premix exTaq kit (Takara). The endogenous gene *Ser1* was used as a control and the primers used are summarized in Supplementary Table 1.

### Protein analysis of transfected cells, middle silk gland, and cocoons of transgenic silkworm

Whole cell proteins in the SF9, BmE and BmNS cells were extracted using RIPA lysis buffer (Beyotime) that included EDTA-free protease inhibitor cocktail (Sigma)^8^. Whole proteins from the middle silk gland were extracted in PBS solution (135 mM NaCl, 2.7 mM KCl, 1.5 mM KH_2_PO_4_, and 8 mM K_2_HPO_4_, pH 7.2) from pieces of the middle silk glands, which were dissected from three randomly positive silkworms and incubated at 4 °C overnight. Whole cocoon proteins were extracted from 20 mg cocoon powder of ten randomly selected cocoons with 1 ml urea solution (8 M urea, 25 mM Tris-HCl, pH 7.0) at 80 °C for 30 min^22^. Then, all protein samples were centrifuged at 17,900 g for 5 min to collect supernatants. After measuring protein concentrations using an Enhanced BCA Protein Assay Kit (Beyotime), samples were subjected to 12% SDS–PAGE and analyzed by Coomassie brilliant blue staining and western blotting. By western blotting, primary antibodies—anti-GFP and anti-Red, anti-FGF1 and anti-HSA, anti-VgR-F or one control primary antibody, β-tubulin, were incubated with the membranes and then with a secondary anti-rabbit IgG antibody. Finally, bands on the membranes were visualized using an ECL Western Blotting Detection System (Amersham Biosciences). Images were recorded by autoexposure for a few seconds using a Chemiscope Series (Clinx science instruments). Experiments were carried out in triplicate.

### Estimation of 2A cleavage efficiency

Cleavage efficiency of the 2A self-cleaving peptide was estimated as described^8^ previously using the following formula: cleavage efficiency = cleaved form/(cleaved form + uncleaved form). The cleaved and uncleaved forms were normalized to β-tubulin. The amounts of each form were calculated based on band intensity on western blots measured using ImageJ software. P-values were determined by two-tailed Student’s *t*-test (n = 3).

### Imaging

For cell imaging, cultured SF9, BmE, and BmNS cells were directly imaged with an Olympus SZX12 fluorescent stereomicroscope (Olympus). Exposure times were 15 ms for DsRed and 100 ms for EGFP after excitation. For the middle silk gland on the seventh day in the fifth stage larva, fluorescence signals were also observed using an Olympus SZX12 fluorescent stereomicroscope and exposure times were 30 ms for DsRed and 80 ms for EGFP after excitation.

## Additional Information

**How to cite this article**: Wang, Y. *et al.* 2A self-cleaving peptide-based multi-gene expression system in the silkworm *Bombyx mori*. *Sci. Rep.* **5**, 16273; doi: 10.1038/srep16273 (2015).

## Supplementary Material

Supplementary Information

## Figures and Tables

**Table 1:** Length and cleavage efficiency of six type 2A self-cleaving peptides.

2A self-cleaving peptide	P2A	T2A	E2A	F2A	BmCPV2A	BmIFV2A
Length^1^	22	21	23	25	33	33
Cleavage efficiency^2^	100%	90%~97%	99%~100%	91%~98%	95%~99%	17%~70%

^1^indicated the amino acids number of 2A self-cleaving peptides containing GSG spacer used in this study.

^2^indicated the cleavage efficiency of 2A self-cleaving peptides which cleave the DsRed-2A-EGFP fusion gene in Sf9, BmNS and BmE cells.

## References

[b1] Xia Q. *et al.* A draft sequence for the genome of the domesticated silkworm (Bombyx mori). Science 306, 1937–1940 (2004). 15591204 10.1126/science.1102210

[b2] Xia Q. *et al.* Complete resequencing of 40 genomes reveals domestication events and genes in silkworm (Bombyx). Science 326, 433–436 (2009). 19713493 10.1126/science.1176620 PMC3951477

[b3] Ryan M.D., King A.M. & Thomas G.P. Cleavage of foot-and-mouth disease virus polyprotein is mediated by residues located within a 19 amino acid sequence. The Journal of General Virology 72 (Pt 11), 2727–2732 (1991). 1658199 10.1099/0022-1317-72-11-2727

[b4] Donnelly M.L. *et al.* Analysis of the aphthovirus 2A/2B polyprotein ‘cleavage’ mechanism indicates not a proteolytic reaction, but a novel translational effect: a putative ribosomal ‘skip’. The Journal of General Virology 82, 1013–1025 (2001). 11297676 10.1099/0022-1317-82-5-1013

[b5] Atkins J.F. *et al.* A case for “StopGo”: reprogramming translation to augment codon meaning of GGN by promoting unconventional termination (Stop) after addition of glycine and then allowing continued translation (Go). Rna 13, 803–810 (2007). 17456564 10.1261/rna.487907 PMC1869043

[b6] Szymczak A.L. *et al.* Correction of multi-gene deficiency *in vivo* using a single ‘self-cleaving’ 2A peptide-based retroviral vector. Nature Biotechnology 22, 589–594 (2004). 10.1038/nbt957 15064769

[b7] Luke G.A. *et al.* Occurrence, function and evolutionary origins of ‘2A-like’ sequences in virus genomes. The Journal of General Virology 89, 1036–1042 (2008). 18343847 10.1099/vir.0.83428-0 PMC2885027

[b8] Minskaia E., Nicholson J. & Ryan M.D. Optimisation of the foot-and-mouth disease virus 2A co-expression system for biomedical applications. BMC Biotechnology 13, 67 (2013). 23968294 10.1186/1472-6750-13-67 PMC3765190

[b9] Fang J. *et al.* Stable antibody expression at therapeutic levels using the 2A peptide. Nature Biotechnology 23, 584–590 (2005). 10.1038/nbt1087 15834403

[b10] Simmons A.D. *et al.* Local secretion of anti-CTLA-4 enhances the therapeutic efficacy of a cancer immunotherapy with reduced evidence of systemic autoimmunity. Cancer Immunology, Immunotherapy: CII 57, 1263–1270 (2008). 18236040 10.1007/s00262-008-0451-3 PMC11031020

[b11] Ha S.H. *et al.* Application of two bicistronic systems involving 2A and IRES sequences to the biosynthesis of carotenoids in rice endosperm. Plant Biotechnology Journal 8, 928–938 (2010). 20649940 10.1111/j.1467-7652.2010.00543.x

[b12] Mikkelsen M.D., Olsen C.E. & Halkier B.A. Production of the cancer-preventive glucoraphanin in tobacco. Molecular Plant 3, 751–759 (2010). 20457641 10.1093/mp/ssq020

[b13] Trichas G., Begbie J. & Srinivas S. Use of the viral 2A peptide for bicistronic expression in transgenic mice. BMC Biology 6, 40 (2008). 18793381 10.1186/1741-7007-6-40 PMC2553761

[b14] Dempsey W.P., Fraser S.E. & Pantazis P. PhOTO zebrafish: a transgenic resource for *in vivo* lineage tracing during development and regeneration. PloS one 7, e32888 (2012). 22431986 10.1371/journal.pone.0032888 PMC3303793

[b15] Deng W. *et al.* Use of the 2A peptide for generation of multi-transgenic pigs through a single round of nuclear transfer. PloS one 6, e19986 (2011). 21603633 10.1371/journal.pone.0019986 PMC3094386

[b16] Daniels R.W., Rossano A.J., Macleod G.T. & Ganetzky B. Expression of multiple transgenes from a single construct using viral 2A peptides in Drosophila. PloS One 9, e100637 (2014). 24945148 10.1371/journal.pone.0100637 PMC4063965

[b17] Tian Y. *et al.* Expression of 2A peptide mediated tri-fluorescent protein genes were regulated by epigenetics in transgenic sheep. Biochemical and Biophysical Research Communications 434, 681–687 (2013). 23603255 10.1016/j.bbrc.2013.04.009

[b18] Holst J., Vignali K.M., Burton A.R. & Vignali D.A. Rapid analysis of T-cell selection *in vivo* using T cell-receptor retrogenic mice. Nature Methods 3, 191–197 (2006). 16489336 10.1038/nmeth858

[b19] Kim J.H. *et al.* High cleavage efficiency of a 2A peptide derived from porcine teschovirus-1 in human cell lines, zebrafish and mice. PloS One 6, e18556 (2011). 21602908 10.1371/journal.pone.0018556 PMC3084703

[b20] Lin Y. *et al.* Vitellogenin receptor mutation leads to the oogenesis mutant phenotype “scanty vitellin” of the silkworm, Bombyx mori. The Journal of Biological Chemistry 288, 13345–13355 (2013). 23515308 10.1074/jbc.M113.462556 PMC3650373

[b21] Wang F. *et al.* Advanced silk material spun by a transgenic silkworm promotes cell proliferation for biomedical application. Acta Biomaterialia 10, 4947–4955 (2014). 24980060 10.1016/j.actbio.2014.06.031

[b22] Wang F. *et al.* An optimized sericin-1 expression system for mass-producing recombinant proteins in the middle silk glands of transgenic silkworms. Transgenic Research 22, 925–938 (2013). 23435751 10.1007/s11248-013-9695-6

[b23] Horn C. & Wimmer E.A. A versatile vector set for animal transgenesis. Development Genes and Evolution 210, 630–637 (2000). 11151300 10.1007/s004270000110

[b24] de Felipe P., Luke G.A., Brown J.D. & Ryan M.D. Inhibition of 2A-mediated ‘cleavage’ of certain artificial polyproteins bearing N-terminal signal sequences. Biotechnology Journal 5, 213–223 (2010). 19946875 10.1002/biot.200900134 PMC2978324

[b25] Ho S.C. *et al.* Comparison of internal ribosome entry site (IRES) and Furin-2A (F2A) for monoclonal antibody expression level and quality in CHO cells. PloS one 8, e63247 (2013). 23704898 10.1371/journal.pone.0063247 PMC3660568

[b26] Hurh S. *et al.* Expression analysis of combinatorial genes using a bi-cistronic T2A expression system in porcine fibroblasts. PloS one 8, e70486 (2013). 23922997 10.1371/journal.pone.0070486 PMC3726604

[b27] Jiang L. *et al.* Resistance to BmNPV via overexpression of an exogenous gene controlled by an inducible promoter and enhancer in transgenic silkworm, Bombyx mori. PloS One 7, e41838 (2012). 22870254 10.1371/journal.pone.0041838 PMC3411602

[b28] Jiang L. *et al.* Resistance to Bombyx mori nucleopolyhedrovirus via overexpression of an endogenous antiviral gene in transgenic silkworms. Archives of Virology 157, 1323–1328 (2012). 22527866 10.1007/s00705-012-1309-8

[b29] Jiang L. *et al.* Identification of a midgut-specific promoter in the silkworm Bombyx mori. Biochemical and Biophysical Research Communications 433, 542–546 (2013). 23524268 10.1016/j.bbrc.2013.03.019

[b30] Jiang L. & Xia Q. The progress and future of enhancing antiviral capacity by transgenic technology in the silkworm Bombyx mori. Insect Biochemistry and Molecular Biology 48, 1–7 (2014). 24561307 10.1016/j.ibmb.2014.02.003

[b31] Wang F. *et al.* Highefficiency system for construction and evaluation of customized TALENs for silkworm genome editing. Molecular Genetics and Genomics: MGG 288, 683–690 (2013). 24077893 10.1007/s00438-013-0782-4

[b32] Thomas J.L., Da Rocha M., Besse A., Mauchamp B. & Chavancy G. 3×P3-EGFP marker facilitates screening for transgenic silkworm Bombyx mori L. from the embryonic stage onwards. Insect Biochemistry and Molecular Biology 32, 247–253 (2002). 11804796 10.1016/s0965-1748(01)00150-3

[b33] Tamura T. *et al.* Germline transformation of the silkworm Bombyx mori L. using a piggyBac transposon-derived vector. Nature Biotechnology 18, 81–84 (2000). 10.1038/71978 10625397

[b34] Wang F. *et al.* Remobilizing deleted piggyBac vector post-integration for transgene stability in silkworm. Molecular genetics and genomics: MGG 290, 1181–1189 (2015). 25589404 10.1007/s00438-014-0982-6

