# Supplementary material for: 2A self-cleaving peptide-based multi-gene expression system in the silkworm Bombyx mori
Source: Sci Rep. 2015 Nov 5;5:16273. doi: 10.1038/srep16273 (PMC4633692; doi:10.1038/srep16273)
Supplement: Supplementary Information [file srep16273-s1.pdf]

2A self-cleaving peptide-based multi-gene expression system in the silkworm *Bombyx mori*

Yuancheng Wang[1,2,+], Feng Wang[1,2,+], Riyuan Wang[1], Ping Zhao[1,2], Qingyou Xia[1,2,*]

[1] State key laboratory of silkworm genome biology, Southwest University, Chongqing, China

[2] College of biology and technology, Southwest University, Chongqing, China

[+] These authors contribute equally to this work

* Corresponding author

E-mail: xiaqy@swu.edu.cn

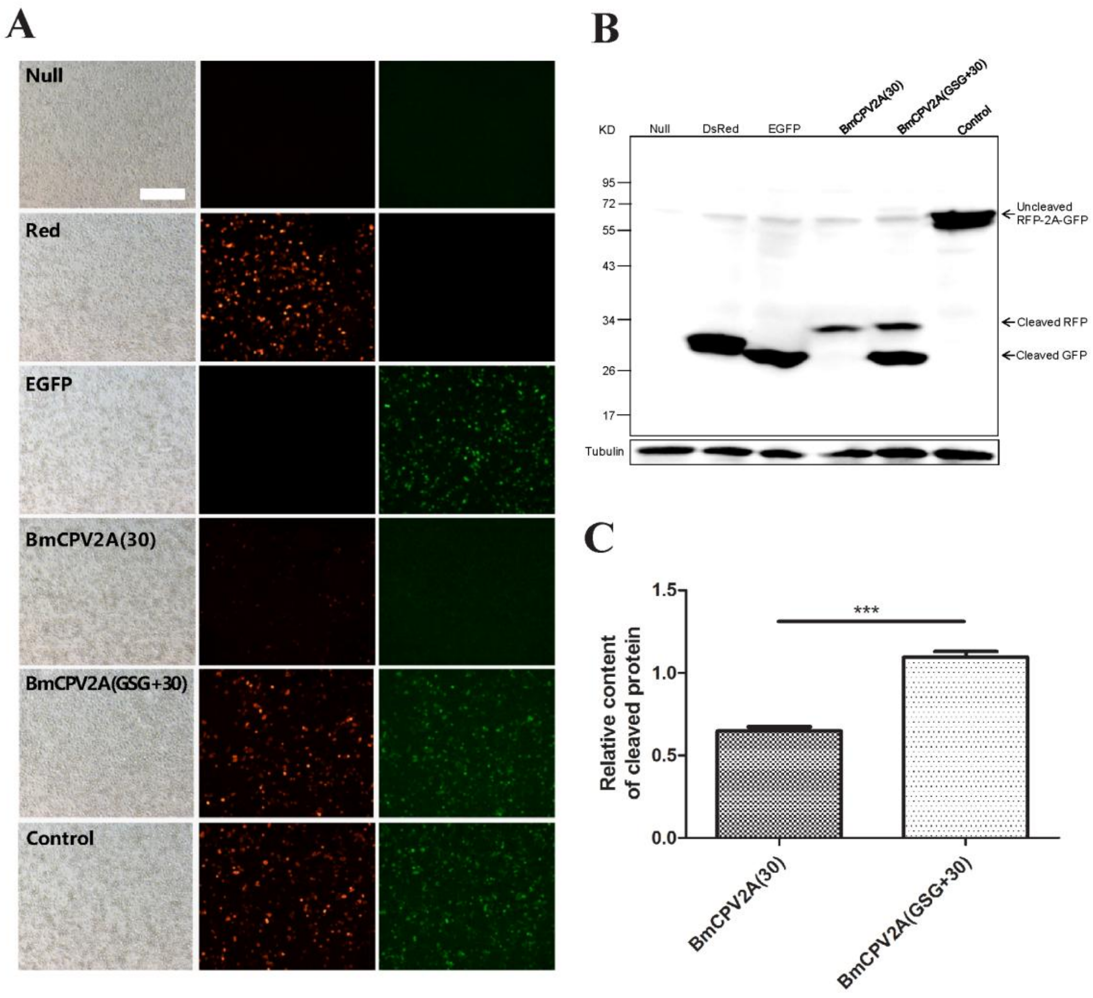

Supplementary information, Figure S1. GSG spacer improved the cleavage efficiency of 2A self-cleaving peptide in BmNS cell. (A) The results of fluorescent signal post-transfection in the BmNs cells. (B) Protein analysis of samples from the BmNS cells. (C) Cleavage efficiency analysis between BmCPV2A(30) and BmCPV2A(GSG+30) in the BmNS cells. Scale bar, 400um.

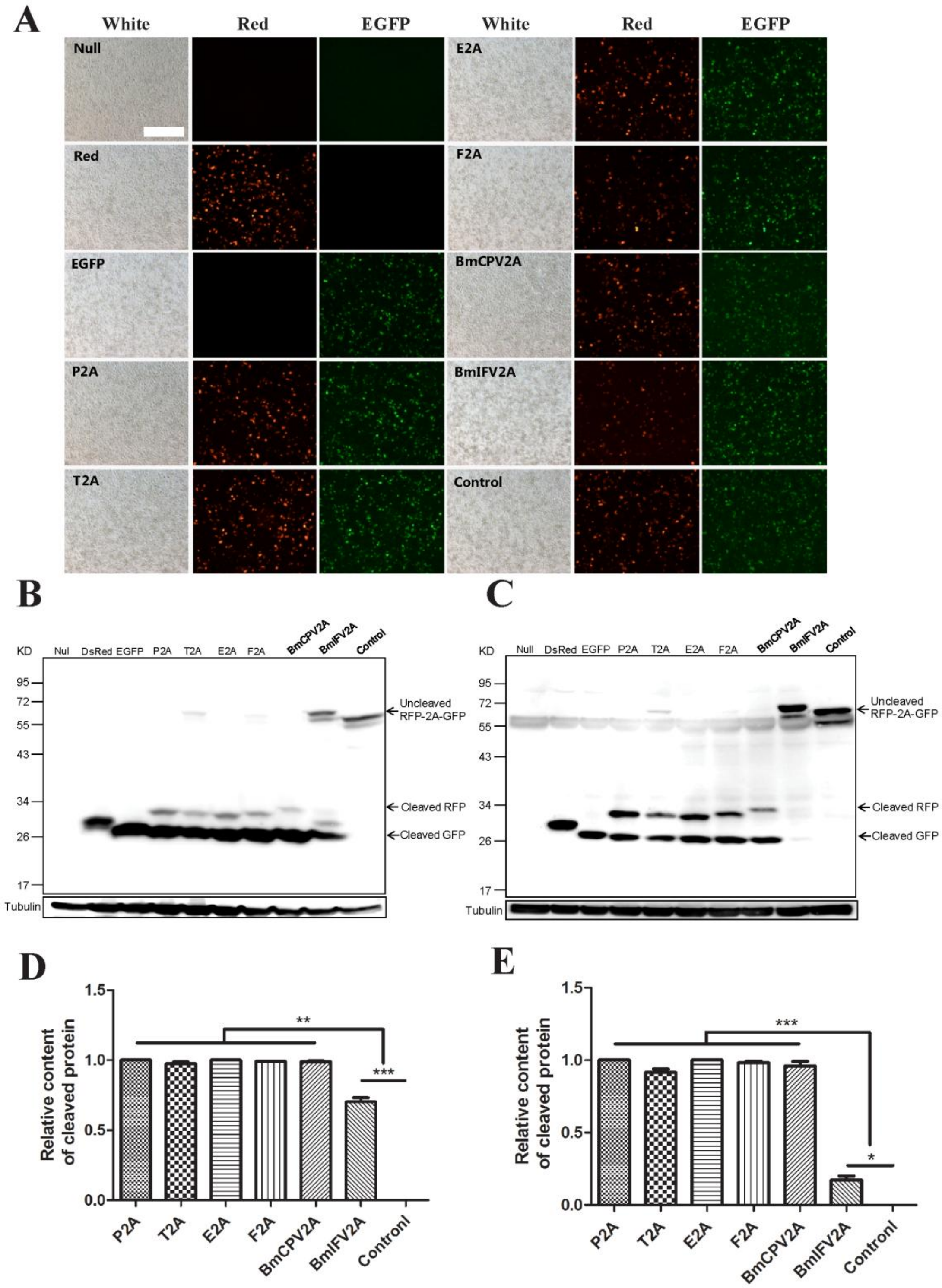

Supplementary information, Figure S2. P2A-GSG and E2A-GSG cleave DsRed-2A-EGFP fusion gene efficiently in the BmE, BmNS cells. (A) The results of fluorescent signal post-transfection in the BmNS cell. (B) and (C) Protein analysis of samples from the BmNS and BmE cells. (D) and (E) Cleavage efficiency analysis of six type 2A self-cleaving peptides in the BmNS and BmE cells. Scale bar, 400 um.

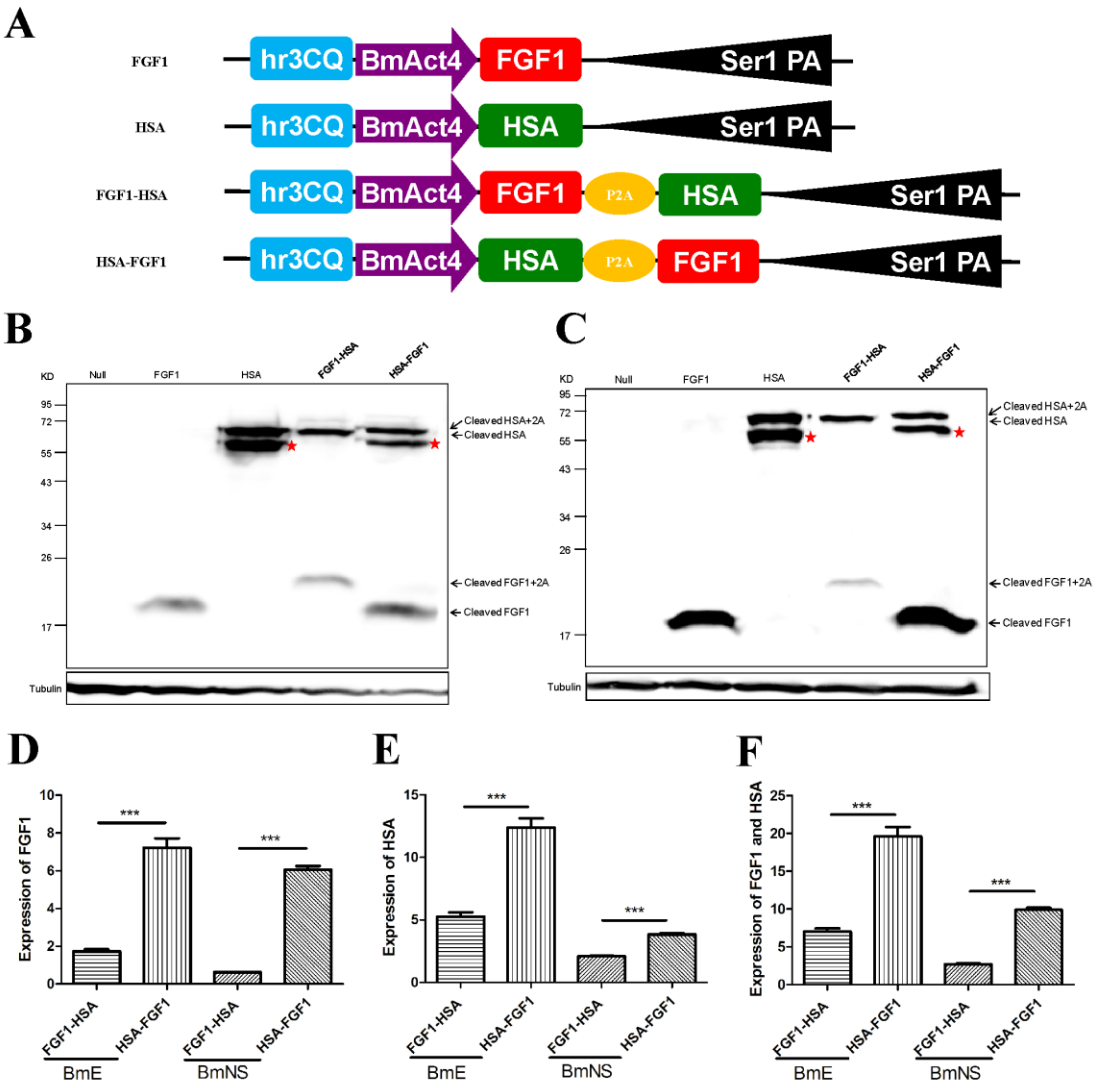

Supplementary information, Figure S3. P2A-GSG could cleave the artificial FGF1-2A-HSA and HSA-2A-FGF1 fusion genes. (A) Schematic diagram of the FGF1, HSA, FGF1-2A-HSA and HSA-2A-FGF1 expression vectors. GSG spacer was added at the N-terminus of P2A. (B) and (C) Protein analysis of samples from the BmE and BmNS cells. In western blotting, two mixed primary antibodies (anti-HSA antibody plus anti-FGF1 antibody) were used, and the second antibody was anti-rabbit IgG. The bands with asterisks indicated the degraded HSA or HSA-2A fusion proteins. (D), (E), (F) respectively indicated the comparison quantitatively of the expression of FGF1, HSA and FGF1plus HSA in the BmE, BmNS cell.

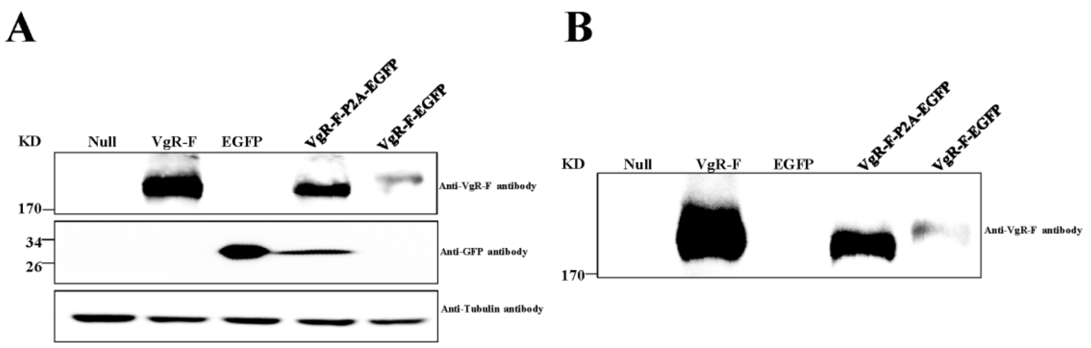

Supplementary information, Figure S4. P2A-GSG cleaved the VgR-F-P2A-EGFP fusion gene in the BmE cell. (A) Protein analysis of samples from the BmE cells. (B) Protein analysis of samples from the culture medium of BmE cells.

Supplementary information, Table S1. Primers used in this study.

Primer	Sequence（5’-3’）
BamHI-DsRed Forward[1,2,3,4,5,6,7]	CTAGGGATCCATGGTGCGCTCCTCCAAGAA
EGFP-NotI Reverse[1,2,3,4,5,6,7]	CTAGGCGGCCGCTTACTTGTACAGCTCGTCCATGCC
DsRed-P2A Reverse[1]	AGGTCCAGGGTTCTCCTCCACGTCTCCAGCCTGCTTCAGCAGGCTGAAGTTAGTAGCTCCGCTTCCCAGGAACAGGTGG
EGFP-P2A Forward[1]	GGAAGCGGAGCTACTAACTTCAGCCTGCTGAAGCAGGCTGGAGACGTGGAGGAGAACCCTGGACCTGTGAGCAAGGGCG
DsRed-T2A Reverse[2]	AGGTCCAGGATTCTCCTCGACGTCACCGCATGTTAGCAGACTTCCTCTGCCCTCTCCGCTTCCCAGGAACAGGTGG
EGFP-T2A Forward[2]	GGAAGCGGAGAGGGCAGAGGAAGTCTGCTAACATGCGGTGACGTCGAGGAGAATCCTGGACCTGTGAGCAAGGGCG
DsRed-E2A Reverse[3]	AGGTCCAGGGTTGCTCTCAACATCTCCAGCCAATTTCAAGAGAGCATAATTAGTACACTGTCCGCTTCCCAGGAACAGGTGG
EGFP-E2A Forward[3]	GGAAGCGGACAGTGTACTAATTATGCTCTCTTGAAATTGGCTGGAGATGTTGAGAGCAACCCTGGACCTGTGAGCAAGGGCG
DsRed-F2A Reverse[4]	AGGTCCAGGGTTGGACTCCACGTCTCCCGCCAACTTGAGAAGGTCAAAATTCAAAGTCTGTTTCACTCCGCTTCCCAGGAACAGGTGG
EGFP-F2A Forward[4]	GGAAGCGGAGTGAAACAGACTTTGAATTTTGACCTTCTCAAGTTGGCGGGAGACGTGGAGTCCAACCCTGGACCTGTGAGCAAGGGCG
DsRed-BmCPV2A Reverse[5]	GCACAACTTTAGTAGGTCATAATTAGAGCGAAAAACGTCCTGCTGGAAATCGAACGCTGTTCTTCCGCTTCCCAGGAACAGGTGG
EGFP-BmCPV2A Forward[5]	CAGCAGGACGTTTTTCGCTCTAATTATGACCTACTAAAGTTGTGCGGTGATATCGAGTCTAATCCTGGACCTGTGAGCAAGGGCG
DsRed-BmIFV2A Reverse[6]	ACGAATCAATTCATCCTCAATCTTCGCCCTCGTCAGAGTCCGCGCGACATTACCAATTGAGGGTCCGCTTCCCAGGAACAGGTGG
EGFP-BmIFV2A Forward[6]	GCGCGGACTCTGACGAGGGCGAAGATTGAGGATGAATTGATTCGTGCAGGAATTGAATCAAATCCTGGACCTGTGAGCAAGGGCG
DsRed-Control Reverse[7]	ACGGCCATTAACGTCACCACAAGTAGAACCACGTCCACCGCCACCCAGGAACAGGTGG
EGFP-Control Forward[7]	GGTGGCGGTGGACGTGGTTCTACTTGTGGTGACGTTAATGGCCGTGTGAGCAAGGGCG
BamHI-FGF1 Forward[8]	CTCTGAGGATCC ATGTTCAACTTACCTCCAGGCAA
NotI-FGF1 Reverse[8]	ATGCTGAGCGGCCGCTTAGAGACCGAGTGCTGCTTGA
FGF1-2A Reverse [8]	CCTCGACGTCACCGCATGTTAGCAGACTTCCTCTGCCCTCTCCGCTTCCTCGCTTTGCTCGGTCAGATGATACGGGCAGC
HSA-2A Forward[8]	AGCGGAGAGGGCAGAGGAAGTCTGCTAACATGCGGTGACGTCGAGGAGAATCCTGGACCTGATGCTCATAAGTCGGAAGT

BamHI-HSA Forward[9]	CTCTGAGGATCCATGGATGCTCATAAGTCGGAAGT
NotI-HSA Reverse[9]	ATGCTGAGCGGCCGCTTAGTCAGATGATACGGGCAGC
HSA-2A Reverse[9]	CCTCGACGTCACCGCATGTTAGCAGACTTCCTCTGCCCTCTCCGCTTCCTCGCTTTGCTCGGAGACCGAGTGCTGCTTGA
FGF1-2A Forward[9]	AGCGGAGAGGGCAGAGGAAGTCTGCTAACATGCGGTGACGTCGAGGAGAATCCTGGACCTTTCAACTTACCTCCAGGCAA
XhoI-P2A-EGFP-Forward[10]	CTGACTCGAG CGCCACCACCTGTTCCTG
XhoI -EGFP-Forward[11]	CTGAGCGGCCGCTTACTTGTACAGCTCGTCCATGCC
NotI-P2A-ECFP-Reverse[12]	CTGACTCGAGATGGTGAGCAAGGGCG
Realtime-DsRed-Forward[13]	CGCCACCATTTGTTCCTC
Realtime-EGFP-Reverse[14]	TGAGGGCAGCCAAAGCAA

[1-7] indicated the primers used to construct the fusion gene Red-P2A-EGFP, Red-T2A-EGFP, Red-E2A-EGFP, Red-F2A-EGFP, Red-BmCPV2A-EGFP and Red-BmIFV2A-EGFP, respectively. [8-9] indicated the primers used to construct fusion gene FGF1-P2A-HSA, HSA-P2A-FGF1, respectively. [10-12] indicated the primers used to construct fusion gene VgR-F-P2A-EGFP and VgR-F-P2A-EGFP. [13-14] indicated the primers used to detect the expression of fusion gene spDsRed-P2A-spEGFP in mRNA level by real time PCR .
